# Clinical and Epidemiological Characteristics of Sporotrichosis in a Reference Center of Uruguay

**DOI:** 10.3390/jof8030322

**Published:** 2022-03-21

**Authors:** Elisa Cabeza, Annie Arrillaga, Lucía Dalcín, Mauricio Carbia, Zaida Arteta, Patricia Perera

**Affiliations:** 1 Cathedra of Infectious Diseases, Faculty of Medicine, University of the Republic, Montevideo 11300, Uruguay; luciadalcinnovelli@gmail.com (L.D.); zaidaarteta@gmail.com (Z.A.); 2 Mycology Laboratory, Department of Parasitology and Mycology, Institute of Hygiene, Faculty of Medicine, University of the Republic, Montevideo 11300, Uruguay; anniearrillaga@gmail.com (A.A.); ppererah@gmail.com (P.P.); 3 Department of Clinical Laboratory, Faculty of Medicine, University of the Republic, Montevideo 11300, Uruguay; mcarbia@gmail.com

**Keywords:** sporotrichosis, *Sporothrix* spp., implantation mycosis

## Abstract

Background: Sporotrichosis is a fungal implantation disease of subacute/ chronic course caused by species of the dimorphic fungus *Sporothrix* spp. This infection usually develops after traumatic inoculation of contaminated soil, plants or organic material contaminated by *Sporothrix* spp. conidia into skin or mucosa. The objective of this work is to contribute to the knowledge of sporotrichosis in Uruguay by providing a report of a series of cases diagnosed in a reference center. Methods: We conducted a retrospective, observational, descriptive and cross-sectional study of cases of sporotrichosis diagnosed in the last 38 years. Results: In the period analyzed, 157 cases of sporotrichosis were diagnosed, 152 of those corresponded to male patients. The most frequent clinical presentation was nodular lymphatic in 120 patients. In relation to epidemiological antecedents, 128 patients had been scratched by armadillos during hunting. Conclusions: Sporotrichosis in Uruguay is a sporadic disease with a clear seasonal pattern related to particular social practices, such as hunting armadillos. Related to this practice, the affectation is greater in males and in young adults.

## 1. Introduction

Sporotrichosis is a fungal implantation disease of subacute/ chronic course caused by species of the dimorphic fungus *Sporothrix schenckii* complex [1,2,3,4]. This disease has a worldwide distribution with high incidence in Latin America, India, Japan, China and South Africa [5,6]. It is a cosmopolitan disease and particularly endemic in temperate zones with humid climates [7,8]. It is considered the most frequent subcutaneous mycosis in Latin America [9,10].

It is characterized by nodular lesions in the skin and in the subcutaneous tissue that subsequently ulcerates, mainly affecting the lymphocutaneous system, but rarely affecting other organs [1,11,12,13,14,15]. This infection usually develops after the traumatic inoculation of contaminated soil, plants, and organic material contaminated by *Sporothrix* spp. conidia into skin or mucosa [11,12,13,14,15,16]. Infection with this fungus is related to areas of the body exposed to trauma [17,18]. Historically, sporotrichosis was known as the “gardener’s mycosis”, classically associated with soil manipulation activities, whether due to occupational or leisure reasons [9,19,20].

Although Sapronotic transmission was historically the most common source of human sporotrichosis, zoonotic infections have become increasingly common [21]. In Uruguay, the transmission was reported to be related to armadillo hunting and more recently in Brazil and Argentina related to cats [9,22,23,24]. Species of the *Sporothrix schenckii* complex are highly successful mammal pathogens, including *S. brasiliensis*, *S. schenckii sensu stricto*, *S. globosa*, and *S. luriei*, agents of human and animal sporotrichosis [16]. Particularly, *S. brasiliensis* is reported as an emerging fungal pathogen with cat-to-human (zoonotic) and cat-to-cat/dog transmission (enzootic) and epidemic and epizootic potential [21,24]. Sporotrichosis is an emergent disease and, over the past two decades, the incidence of zoonotic sporotrichosis has risen, particularly in Brazil [16]. Scratches and bites or contact with the exudate of cutaneous lesions of infected cats transmits sporotrichosis, in the great majority of the cases by *S. brasiliensis* [2,3,24,25,26].

Some authors hypothesized that the cats’ infection may occur via an inhalation route, as cats have nasal mucosal lesions and the *Sporothrix* spp. were isolated from nasal cavity and lungs. However, healthy cats have a minor role in sporotrichosis transmission because it is believed that it is related to a low charge of spores of *Sporothrix* spp. in the oral cavity and claws of healthy cats [24,25,26,27,28,29].

The epidemiological profile is mainly characterized by children, elderly, and women, because these groups usually have direct and more frequent contact with these animals [9,30].

Recently, genetic sequencing studies confirmed that *S. schenckii* is a complex composed of cryptic species: *S. albicans, S. brasiliensis, S. globosa, S. luriei, S. mexicana,* and *S. schenckii* [17,31,32,33]. *S. brasiliensis* and *S. schenckii* are common in Brazil, *S. mexicana* is present in Mexico, and *S. globosa* is common in countries such as China, India, Japan, the USA, Spain and Italy [17,34]. Interestingly, it arises that the crypticas species have a different pathogenic potential and *S. schenckii* causes a benign chronic subcutaneous mycosis, *S. brasiliensis* is highly virulent, and *S. globosa* causes mainly fixed cutaneous lesions [5,35,36]. The aim of this work is to contribute to the knowledge of sporotrichosis in Uruguay by reporting of a series of cases diagnosed in a reference center.

## 2. Materials and Methods

We conducted a retrospective, observational, descriptive and cross-sectional study of cases of sporotrichosis diagnosed in the last 38 years (1 January 1983 to 31 December 2020), using a database of patients diagnosed in this period in the outpatient clinics of parasitic and fungal diseases of the Department of Parasitology and Mycology of the Faculty of Medicine, University of the Republic—Uruguay.

This outpatient clinic is a mycology and parasitology reference center for the public and private sub sectors of the health system, with mostly patients of the public subsector assisted here.

We highlight that the population included in this work were referred from specialist doctors and generalist doctors from different assistant centers. For this reason, the cases remitted here have a more complex diagnosis and/or in the referring center and parasitology and mycology specialists are not included. Thus, the data do not show a reflection of sporotrichosis in the general population. 

This review was constructed from the records of the outpatient assistance of the parasitology and mycology and the cases were enrolled in a SPSS 22 database. The clinical and analytical data are associated with the collection of direct exams and cultures with an autogenerated number. 

The diagnosis was made by direct study of the secretion of the ulcerated lesions on fresh material, by making appositional smears for Gram staining, and confirmed by culture in Sabouraud media at 28 °C of the samples obtained from the ulcerated lesions. Genus identification was performed by visualizing the micromorphology of the colonies.

All patients with a diagnosis of sporotrichosis, confirmed with cultures in Sabouraud agar medium at 28 °C, were included in this study.

In reference to the clinical data we collected, we recorded age, biological sex, epidemiological history, origin and area of residence, occupation, month of the year in which the diagnosis was made, clinical manifestations, immune status, biological sample from which the diagnosis, findings on direct mycological examination and antimicrobial treatments received.

### Ethical Aspects

This case report, including the collection and evaluation of the patient’s protected medical information, was conducted in accordance to the Personal Data Protection Law N 18331 of Uruguay of 2008 and adhered to the ethical principles of the Medical World Association for medical research involving human subjects described in the Declaration of Helsinki as amended in 2013. The ethical review and approval of this study was waived because the clinical records did not present patronymic data associated with the historical database, and the patients could not be identified. We believe this report will benefit the diagnosis and management of future patients with sporotrichosis.

## 3. Results

In the period analyzed, 157 cases of sporotrichosis were diagnosed, 152 (97.0%) of which corresponded to male patients and only four of which (2.5%) corresponded to female patients (Table 1).

**Figure 1:**
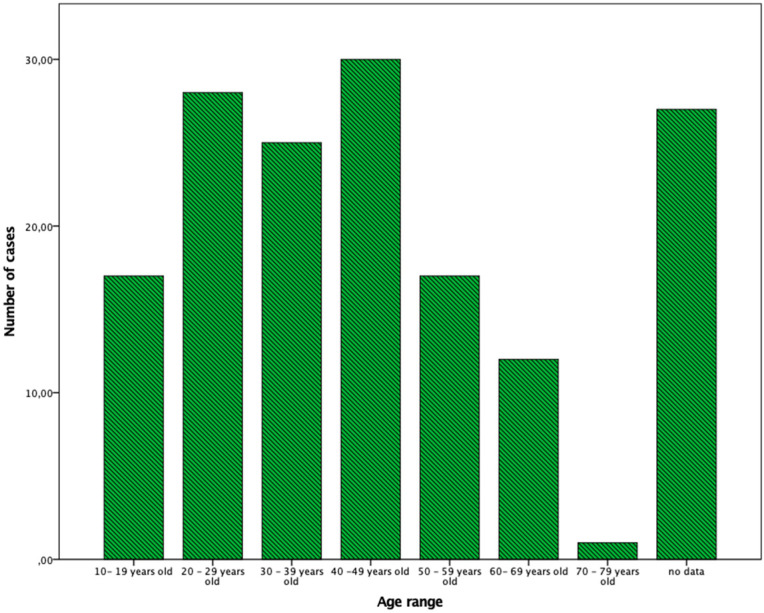
Detailed distribution of cases according to age ranges. On the Y axis the number of cases is detailed, on the X axis the eta range is specified.

The age range was between 13 and 79 years old, with a media of 30.8 ± 12.3 years and 54.1% of the patients were between 13 and 49 years old (Figure 1).

Regarding the occupation of the patients, 63 patients had jobs not related to possible exposure to sporotrichosis (merchants, salesmen, employees, administrative), 24 patients were rural workers, six patients were gardeners, four constructors; 15 of them were high school students, seven were retired, three were military officers and occupational data is not available in 35 cases (Table 1).

**Figure 2:**
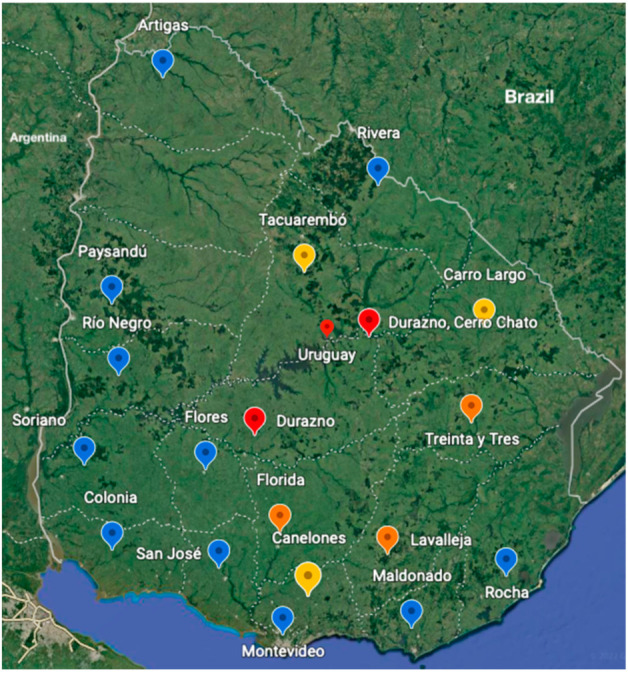
Distribution of cases of sporotrichosis in Uruguay. A color scale is used, blue corresponds to provinces with 5 cases or less, provinces in yellow between 6–10 cases, provinces in orange between 11–20 cases, red province with the highest number of cases (35 cases).

In relation to the place of acquisition of sporotrichosis, the majority (n = 132, 84.1%) of the patients acquired it in the provinces, linked to rural areas. The distribution of the majority of sporotrichosis cases is concentrated in the south central region of Uruguay. The province with the highest number of cases was Durazno, with 34 cases. The following provinces in frequency were Lavalleja and Florida with 18 cases each. In the provinces of Treinta y Tres, we registered 13 cases, in Tacuarembó 10 cases, in Cerro Largo and Canelones nine cases each, five cases in Montevideo, four cases in Rocha, in Paysandú and Flores three cases were registered each, in Rio Negro and Colonia two cases were recorded for each and in the departments of Rivera, Artigas, Maldonado and Soriano one case was recorded in each province. In 20 cases, data on the place of acquisition of the sporotrichosis was not obtained. (Table 1, Figure 2).

According to the location of the entrance door, 115 patients with lesions to the upper limbs (73.2%), 16 patients to the lower limbs (10.2%) and six cases (3.8%) in other locations (neck, chest and abdomen). In 20 cases (12.7%), the anatomical site of the lesion was not recorded in the registry (Table 2).

**Figure 3:**
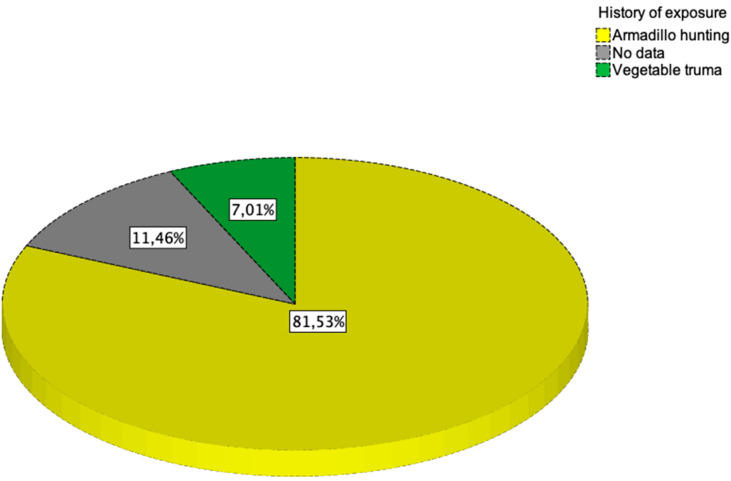
Detailed distribution according to history of exposure. Yellow represents the percentage of total cases with a history of trauma from hunting armadillos. Green represents the percentage of total cases with a history of trauma with vegetables. In gray it is represented the percentage of total cases which does not have a traumatic antecedent or the patient does not know how to specify.

In relation to epidemiological antecedents, 128 patients (81.5%) had scratches from armadillos obtained during hunting, while in 11 cases (7.0%) injuries from plants were reported or a different cause was identified. A total of 18 cases (11.5%) did not have a traumatic antecedent or the patient did not know how to describe it (Figure 3).

**Figure 4:**
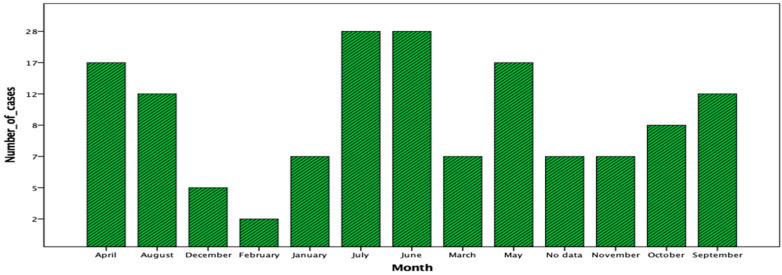
Detailed cases distribution in months of year. The X axis specifies the month of the year in which the diagnosis was made, and the Y axis details the number of cases of sporotrichosis diagnosed.

Although cases are reported throughout the year, there are concentrations of them between April and September, with 114 cases being found in this period (72.6%) (Figure 4).

The most frequent clinical presentation was nodular lymphatic in 120 patients (76.4%), fixed forms were in 30 cases (19.1%) and it was not recorded in seven cases (4.5%) (Table 2).

In a direct mycology study, asteroid bodies were found in 82 patients (52.3%), while in the other 75 patients they were not (47.7%) (Table 2). We highlight that all samples with asteroid bodies in the direct mycology study developed colonies compatible with *Sporothrix* spp. 

At the moment of outpatient assistance, 32 patients (20.4%) received treatment with antibiotics, 17 (10.8%) with antifungals and one patient with both. The antibiotics were trimethoprim sulfamethoxazole in 12 cases and cephradine in 20 cases. All the patients treated with antifungals received itraconazole. The patient with both treatments received cephradine and itraconazole.

## 4. Discussion

Although the cases presented here were distributed across all age groups, there is a clear predominance among male young adults. In fact, this characteristic of the population is related to the mechanism of acquisition particularly in Uruguay by armadillo hunting [22,23,24]. In other countries, they are more often related to agriculture and floriculture tasks, and therefore predominate in women and children [25,27,30]. Since the late 1990s, sporotrichosis in Rio de Janeiro, Brazil, has become an urban endemic phenomenon, and the epidemic is linked to transmission from infected cats to humans. The high prevalence of cases from the metropolitan area of Rio de Janeiro has created a sporotrichosis belt. The majority are female patients and from poor socioeconomic backgrounds who acquire the disease through domiciliary or professional contact (bite or scratch) with cats infected with sporotrichosis [5,32,36,37].

In most of our patients, sporotrichosis was linked to scratches by armadillos, another less frequent mechanism are scratches by vegetable thorns and contaminated wood splinters. In Uruguay, the acquisition is strongly linked to armadillo hunting, in fact, this epidemiological antecedent was reported historically in our country [22]. However, it cannot be classified as a zoonotic transmission as it occurs in cat–human transmission, since armadillos do not present sporotrichosis. Thus, inoculation occurs through scratches in the manipulation of the armadillos, since the spores of *Sporothrix* spp. are found in their caves. Secondary to this practice, the affectation is greater in males and an average age of young adults. In this way, the anatomical involvement is greater at the level of the upper limbs than the involvement of other locations to a lesser extent.

The most frequent clinical presentation was the lymphangitic-nodular cutaneous form in the upper limbs that appears at an average of 3 months before the microbiological diagnosis. This determines that the patients presented the characteristic clinic with multiple nodules in different evolutionary stages. Fixed forms have been seen in a small number of cases, which requires more time to diagnose.

In Uruguay, the distribution of cases presents a clear predominance in the austral autumn and winter months; linked to the high frequency of armadillo hunting in March and April connected to autumn holidays.

After inoculation, the coldest months of the year arrive, which favors a progression of the disease. The etiological diagnosis is generally late, between 1 to 3 months after the onset of symptoms.

We highlight the importance of the visualization of asteroid bodies in mycological direct study, as this permits shorter times to diagnose with respect to the culture, within just a few minutes after taking of samples [38,39,40]. The observation of microscopic asteroid bodies allows for the prescription of specific treatment in the initial consultation. At the time of microbiological diagnosis, direct visualization of asteroid bodies depends on the quality of the image and the expertise of the team that performs the diagnosis; our center had a good performance in this case. In the present series, asteroid bodies were observed in 52,3% of the cases, less than those reported by other authors, which could be linked to the number of plates studied [39]. A sensitivity of the order of 90% was reported in the context of research work with the study of five live slides; this can hardly be performed in clinical diagnostic laboratories due to the high demand for time that it requires [38,40].

The diagnostic confirmation via the development of colonies compatible with *Sporothrix* spp. in the cultures in cases of clinical suspicion, epidemiological background and positive direct microscopy was consistent with what was expected. However, the confirmation of microbiological diagnosis is always late.

A small percentage of patients were initially treated with antibiotics, which reinforces the concept of the need for broad knowledge of the epidemiology and clinical presentation of sporotrichosis, in order to establish a correct diagnosis and treatment and avoid incorrect exposure to antimicrobials. Until 1986, the indicated treatment was potassium iodide and thermotherapy [41], in the following years itraconazole-based therapy was introduced at a dose of 200 to 400 mg/day from 4 to 6 months depending on the clinical presentation [42,43], in all cases associated with the application of local thermotherapy [41,42]. All patients who received itraconazole responded favorably to treatment at a dose of 200 mg every 12 h for up to 15 days after a resolution of the lesions.

## 5. Conclusions

Sporotrichosis in Uruguay is a sporadic disease with a clear seasonal pattern related to a particular social practice such as hunting armadillos. The epidemiological variation in relation to gender and transmission mechanism opens the possibility in our country of the beginning of other epidemiological mechanisms already described in other countries of the region that have not appeared so far. Regarding the late clinical forms of presentation, the importance of the diagnostic approach with direct mycological study and the confirmation of microbiological diagnosis, which is always late, we strongly recommend consultation with an infectious diseases specialist in a patient with clinical suspected sporotrichosis, with or without an epidemiological history.

## Figures and Tables

**Table 1:** Demographic characteristics of 157 patients with sporotrichosis.

Characteristics	n (%)
Sex	
Male	152 (97.0)
Female	4 (2.5)
Occupation	
Without occupational hazard (driver, dressmaker, housekeeper, teacher, student, administrative, employees, merchants) and retired	85 (54.1)
Rural workers	24 (15.3)
Gardeners	6 (3.8)
Constructor	4 (2.5)
Military officer and Police	3 (1.9)
Non-registered	35 (22.3)
Place of residence	
Durazno	34 (21.6)
Lavalleja	18 (11.5)
Florida	18 (11.5)
Treinta y Tres	13 (8.3)
Tacuarembó	10 (6.4)
Cerro Largo	9 (5.7)
Canelones	9 (5.7)
Montevideo	5 (3.2)
Rocha	4 (2.5)
Paysandú	3 (1.9)
Flores	3 (1.9)
Río Negro	2 (1.3)
Colonia	2 (1.3)
Artigas	1 (0.64)
Rivera	1 (0.64)
Maldonado	1 (0.64)
Soriano	1 (0.64)
Non-registered	20 (12.7)

**Table 2:** Distribution according to lesion site, classification of sporotrichosis and direct mycology study results.

Characteristics	n (%)
Lesion site	
Upper limbs	115 (73.2)
lower limbs	16 (10.2)
other locations (neck, chest and abdomen)	6 (3.8)
Classification sporotrichosis	
Nodular lymphatic	120 (76.4)
Fixed cutaneous	30 (19.1)
Non-registered	7 (4.5)
Direct mycology study	
Asteroid bodies	82 (52.3)
Negative	75 (47.7)

## Data Availability

Not applicable.
